# Supplementary material for: Perisomatic GABAergic synapses of basket cells effectively control principal neuron activity in amygdala networks
Source: eLife. 2017 Jan 6;6:e20721. doi: 10.7554/eLife.20721 (PMC5218536; doi:10.7554/eLife.20721)
Supplement: Figure 1—source data 1. — DOI: http://dx.doi.org/10.7554/eLife.20721.003 [file elife-20721-fig1-data1.docx]

**Figure 1- Source data 1**

**Basic electrophysiological properties of the output synapses of CCKBCs and PVBCs recorded in whole-cell mode**

|  | **CCKBC** | | n= | **PVBC** | | n= | Mann-Whitney test p= |
| --- | --- | --- | --- | --- | --- | --- | --- |
|  | median | IQ range |  | median | IQ range |  |  |
| 1^st^ IPSC amplitude (pA) | 78.20 | 59.21 | 24 | 84.96 | 58.72 | 26 | 0.88 |
| 1^st^ IPSC potency (pA) | 84.65 | 52.00 | 24 | 84.96 | 56.64 | 26 | 0.98 |
| 1^st^ IPSC failure rate | 0.00 | 0.18 | 24 | 0.00 | 0.00 | 26 | 0.062 |
| 1^st^ IPSC 10-90% rise time (ms) | 1.04 | 0.60 | 24 | 0.79 | 0.42 | 26 | **0.004** |
| 1^st^ IPSC decay time constant (ms) | 4.75 | 1.93 | 24 | 4.92 | 2.74 | 24 | 0.88 |
| 1^st^ IPSC latency (ms) | 1.40 | 0.49 | 23 | 0.88 | 0.34 | 24 | **0.001** |
| IPSC peak3/peak1 amplitude | 0.96 | 0.22 | 18 | 0.62 | 0.15 | 24 | **0.00003** |
| 3 IPSC charge (pC) | 1.69 | 1.25 | 22 | 1.38 | 1.22 | 24 | 0.39 |
| 3 IPSP integral (mV*ms) | 124.70 | 105.35 | 15 | 107.50 | 69.90 | 17 | 0.38 |
