# Supplementary material for: Perisomatic GABAergic synapses of basket cells effectively control principal neuron activity in amygdala networks
Source: eLife. 2017 Jan 6;6:e20721. doi: 10.7554/eLife.20721 (PMC5218536; doi:10.7554/eLife.20721)
Supplement: Figure 3—source data 1. — DOI: http://dx.doi.org/10.7554/eLife.20721.008 [file elife-20721-fig3-data1.docx]

**Figure 3- Source data 1**

**Number of the pairs and analyzed contacts at the confocal microscopic level**

| **presynaptic IN type** | **# of analyzed pairs** | **total # of contacts** | **# of contacts on perisomatic region** | **# of contacts on dendrites** |
| --- | --- | --- | --- | --- |
| CCKBC | 38 | 302 | 102 | 200 |
| PVBC | 26 | 283 | 92 | 191 |
