# Supplementary material for: Perisomatic GABAergic synapses of basket cells effectively control principal neuron activity in amygdala networks
Source: eLife. 2017 Jan 6;6:e20721. doi: 10.7554/eLife.20721 (PMC5218536; doi:10.7554/eLife.20721)
Supplement: Figure 3—source data 2. — Data is shown as the number of the analyzed contacts, with the number of verified synapses shown in parentheses. DOI: http://dx.doi.org/10.7554/eLife.20721.009 [file elife-20721-fig3-data2.docx]

**Figure 3- Source data 2**

**Summary table of the contacts analyzed at the electron microscopic level.** Data is shown as the number of the analyzed contacts, with the number of verified synapses shown in parentheses.

| **pair code** | **presynaptic IN type** | **total # of contacts** | **# of contacts on soma** | **# of contacts on dendrites** |
| --- | --- | --- | --- | --- |
| j120102-01 | PVBC | 8 (7) | 5 (5) | 3(2) |
| j120104-03 | PVBC | 8 (7) | 2 (2) | 6 (5) |
| j121122-01 | PVBC | 1 (0) | 0 (0) | 1 (0) |
| j111125-01 | CCKBC | 5 (3) | 4 (2) | 1 (1) |
| j120726-01 | CCKBC | 4 (4) | 0 (0) | 4 (4) |
| j120922-03 | CCKBC | 8 (8) | 5 (5) | 3 (3) |
